# Automated deep learning–based segmentation and volumetric analysis of meningiomas

**DOI:** 10.1007/s00234-026-04152-z

**Published:** 2026-08-20

**Authors:** Daniel de Wilde, Olivier Zanier, Aron Alakmeh, Stefanos Voglis, Ulf C. Schneider, Luca Regli, Carlo Serra, Victor E. Staartjes, Gustav Burström, Raffaele Da Mutten

**Affiliations:** 1 Machine Intelligence in Clinical Neuroscience & Microsurgical Neuroanatomy (MICN) Laboratory, Department of Neurosurgery, Clinical Neuroscience Center, University Hospital Zurich, University of Zurich, Zurich, Switzerland; 2 Department of Oncology, University of Oxford, Oxford, UK; 3 Department of Neurosurgery, Cantonal Hospital Lucerne, Lucerne, Switzerland; 4 Department of Clinical Neuroscience, Karolinska Institutet, Stockholm, Sweden; 5 Capio Spine Center Stockholm, Löwenströmska Hospital, Upplands Väsby, Sweden; 6 Department of Neurosurgery, University Hospital Frankfurt, Frankfurt, Germany

**Keywords:** Artificial intelligence, Deep learning, Meningioma, Segmentation

## Abstract

**Introduction:**

Meningiomas are the most common primary intracranial tumors and are frequently monitored over extended periods. Volumetric assessment typically requires manual segmentation, which is time-consuming and associated with interrater variability. This study aimed to develop and validate a deep learning-based model for the automated segmentation of meningiomas and associated peritumoral edema on preoperative magnetic resonance imaging (MRI).

**Methods:**

We trained a standard nnU-Net deep learning model on contrast-enhanced T1-weighted and FLAIR MRI scans from 100 patients treated at the University Hospital of Zurich. The model was then externally validated on 88 cases from the meningioma SEG-Class dataset from the Cancer Imaging Archive. Segmentation performance was assessed using the Dice similarity coefficient, Jaccard index, and 95th percentile Hausdorff distance.

**Results:**

The model achieved mean Dice scores of 0.87 ± 0.23 for meningioma segmentation and 0.63 ± 0.38 for peritumoral edema in internal cross-validation. On the external validation set, the model achieved scores of 0.86 ± 0.17 for meningioma segmentation and 0.31 ± 0.35 for edema.

**Conclusion:**

The deep learning model demonstrated high accuracy in segmenting meningiomas and modest performance for peritumoral edema. These results support the potential utility of automated segmentation tools in clinical workflows. Future work should focus on validating model performance across larger multi-center datasets.

**Supplementary Information:**

The online version contains supplementary material available at https://doi.org/10.1007/s00234-026-04152-z.

## Introduction

Meningiomas are the most common primary intracranial neoplasms, accounting for approximately 37% of all primary brain tumors [1]. These tumors are typically benign and are thought to originate from meningothelial cells of the arachnoid layers [2, 3]. In recent years, the widespread use of neuroimaging has led to a growing number of incidentally detected meningiomas [4].

Beyond the initial diagnosis, imaging is essential throughout the clinical management of meningiomas, including longitudinal surveillance, preoperative planning, and postoperative follow-up [5]. Given their characteristically slow growth, early detection and consistent volumetric monitoring are essential for informed clinical decision-making and treatment planning [6]. Notably, a rapid or significant increase in tumor volume over time is a key indicator for recommending treatment [5, 7]. In selected cases, advanced tomographic imaging such as somatostatin receptor PET/CT can provide additional information for tumor delineation, radiotherapy planning, and theranostic approaches such as peptide receptor radionuclide therapy (PRRT) [8–10].

Currently, meningioma assessment is primarily based on manual linear measurements to estimate tumor size [11]. However, linear measurements may incompletely capture true tumor burden [11]. Volumetric analysis may provide a more accurate estimate of tumor size, but it is typically performed through manual segmentation using third-party software. This process is labor-intensive and prone to interobserver variability [12].

Advances in machine learning, particularly deep learning (DL) methods such as convolutional neural networks (CNN), have enabled new opportunities for automating the segmentation of medical imaging data [13]. Among these, the nnU-Net framework has emerged as a leading segmentation model. Building upon the U-Net architecture, which consists of CNNs, the nnU-Net adapts automatically to new datasets by configuring preprocessing, training, and postprocessing steps without the need for manual adjustments [14]. The model has demonstrated strong performance across a wide range of segmentation tasks [15–20].

Despite these advances, the application of DL-based segmentation models to meningioma imaging remains relatively limited [21]. Existing models often lack external validation, are trained on single-center datasets, or are not openly available, all of which hinder their translation into clinical practice [21–23]. Furthermore, most existing models segment only the tumor, while omitting peritumoral edema despite its clinical importance [24].

In this study, we developed and validated a multichannel nnU-Net model for automated segmentation of meningiomas and associated peritumoral edema on preoperative MRI. Our aim was to develop the model using an institutional training dataset and evaluate its performance on an independent external validation dataset and to support the translation of automated meningioma segmentation models into clinical practice.

## Methods

### Study overview

We developed a fully automated segmentation pipeline based on a three-dimensional (3D) nnU-Net architecture for the delineation of meningiomas and associated peritumoral brain edema on preoperative magnetic resonance imaging (MRI) scans. Model training and internal validation were performed using an institutional dataset from the University Hospital Zurich. External validation was subsequently conducted on an independent, publicly available dataset from the Cancer Imaging Archive (TCIA).

### Data acquisition

The institutional dataset included 100 patients who underwent surgical resection for meningiomas at the Department of Neurosurgery of the University Hospital Zurich. Data was accessed on the 22nd of January 2023. All cases had histopathologically confirmed diagnoses, regardless of WHO tumor grade. Inclusion criteria required the availability of high-resolution axial MRI sequences, including contrast-enhanced T1-weighted (T1CE), and FLAIR sequences. Patients were excluded if they lacked visible tumors on imaging, did not have preoperative scans, or were under 18 years of age.

To improve generalizability of the model, an independent dataset of 88 patients from the publicly available Meningioma-SEG-CLASS cohort hosted by TCIA was used for external validation [25].

All data were handled in accordance with the Declaration of Helsinki. Ethical approval was obtained from the Cantonal Ethics Committee of Zurich (BASEC ID: 2023 − 00688). Patients consented to research use of their collected medical data. The TCIA data usage policy was considered.

### Image preprocessing

All imaging data were anonymized prior to analysis. DICOM files were converted to the Neuroimaging Informatics Technology Initiative (NIfTI) format to ensure compatibility with deep learning libraries. All scans were resampled to isotropic voxel resolution (1 × 1 × 1 mm^3^) and standardized to a size of 240 voxels for each dimension. Ground truth segmentations for the Zurich dataset were manually annotated in 3D Slicer (version 5.6.2) by trained medical students under resident supervision [26], following a standardized annotation protocol. Ambiguous cases were reviewed with the supervising resident. All segmentations underwent visual quality control in all three anatomical planes.

### Model development

A 3D nnU-Net framework was selected as the base architecture for both tumor and edema segmentation due to its self-configuring mechanism and consistently high performance in medical image segmentation [14]. The model was designed using a multi-channel input layer that allows for simultaneously incorporating T1CE-weighted and FLAIR MRI sequences as input. Automatic planning of the three-dimensional full resolution mode determined resampling to 1.0 × 1.0 × 1.0 mm voxel size, per-modality z-score normalization, a patch-size of 112 × 160 × 128 voxels, and a batch size of 2. Training was performed in five-fold cross-validation (80 training cases, 20 validation cases), with the default trainer. The model was trained for 1000 epochs with an initial learning rate of 0.01 and the default polynomial learning rate scheduler. Data augmentation was also kept at the standard configuration. This included random spatial transformations with rotation and scaling, mirroring along the allowed axes, Gaussian noise, Gaussian blur, multiplicative brightness changes, contrast augmentation, simulated low resolution, and gamma augmentation. All code was executed using Python 3.11.5 with PyTorch 2.1.0 and nibabel 5.1.0 [27–29]. Training and inference were executed on an NVIDIA RTX PRO 6000 Blackwell GPU equipped with 96 GB of memory. For inference, predictions from the five trained folds were averaged. The checkpoint that performed best in validation was chosen instead of the last one. No postprocessing was applied.

### Evaluation metrics

Model performance was evaluated using standard segmentation metrics: the Dice similarity coefficient, Jaccard index, and 95th percentile Hausdorff distance. The Dice and Jaccard indices both quantify overlap between predicted and ground truth segmentations, ranging from 0 (no overlap) to 1 (perfect congruence) [30, 31]. The 95th percentile Hausdorff distance (HD95) quantifies boundary deviation between segmentations while reducing the influence of outliers [32].

### Deployment

To support clinical translation and promote reproducibility, our models are publicly available on GitHub (https://github.com/MICN-Lab/Meningioma_Segmentation), for further validation and refinement (Supplementary Fig. 5). A graphical user interface, together with all necessary libraries, is bundled as an executable to ensure the possibility of translation into clinical practice.

## Results

### Cohorts

A total of 188 patients were included, with 100 patients included from the University Hospital of Zurich and 88 patients included from the Meningioma-SEG-CLASS dataset. Summary demographics and radiological information are provided in Table 1.

**Table 1:** Summary of patient demographics, tumor characteristics, and radiological findings, presented separately for each dataset. Tumor location counts may exceed the total number of patients because larger tumors could contribute to more than one anatomical location

Variable	Cohort	
	Zurich	External
Baseline		
Number of patients	100	88
Age (years), mean ± SD	63 ± 14	55 ± 14
WHO grade		
I	81 (81)	50 (57)
II	14 (14)	38 (43)
III	1 (1)	-
I & II*	1 (1)	-
NA	3 (3)	-
Tumor location		
Convexity, n (%)	26 (26)	19 (21)
Falx and parasagittal, n (%)	20 (20)	16 (18)
Falx, n (%)	16 (16)	-
Parasagittal, n (%)	4 (4)	-
Anterior cranial fossa, n (%)	8 (8)	6 (6)
Olfactory groove, n (%)	8 (8)	-
Middle cranial fossa, n (%)	25 (25)	35 (39)
Sphenoidal wing, n (%)	14 (14)	-
Planum sphenoidale, n (%)	6 (6)	-
Tuberculum sellae, n (%)	4 (4)	-
Cavernous sinus, n (%)	1 (1)	-
Posterior cranial fossa, n (%)	16 (16)	12 (13)
Tentorium, n (%)	10 (10)	-
Petroclival, n (%)	5 (5)	-
Jugular foramen, n (%)	1 (1)	-
Varia, n (%)	5 (5)	2 (2)
Lateral ventricle, n (%)	-	2 (2)
Multiple, n (%)	4 (4)	-
NA, n (%)	1 (1)	-
Tumor volume		
Preoperative		
n	100	88
Tumor		
mean ± SD [ml]	34.140 ± 35.220	37.502 ± 38.943
median (IQR) [ml]	23.415 (34.649)	24.495 (49.968)
PTBE		
mean ± SD [ml]	22.649 ± 35.704	28.053 ± 38.429
median (IQR) [ml]	3.242 (32.757)	11.193 (29.967)
EOR		
STR, n (%)	17 (17)	-
GTR, n (%)	77 (77)	-
NA, n (%)	6 (6)	-
MRI Data		
Number of sequences (two per patient), n (%)	200 (100)	
Vendor		
Siemens, n (%)	102 (51)	
Philips Healthcare, n (%)	62 (31)	
GE Medical Systems, n (%)	36 (18)	
Field Strength		
1 T, n (%)	2 (1)	
1.5 T, n (%)	44 (22)	
3 T, n (%)	154 (77)	
Voxel Dimensions		
Pixel spacing, mean ± SD (mm)	0.50 ± 0.21	
Pixel spacing, median (IQR) (mm)	0.47 (0.06)	
Slice thickness, mean ± SD (mm)	1.55 ± 1.07	
Slice thickness, median (IQR) (mm)	1 (1)	
Pulse Sequence Parameters		
Repetition Time		
mean ± SD (ms)	3382 ± 2566	
median (IQR) (ms)	2631 (3330)	
NA, n (%)	6 (3)	
Echo Time		
mean ± SD (ms)	137 ± 159	
median (IQR) (ms)	50 (130)	
NA, n (%)	0 (0)	
Inversion Time		
mean ± SD (ms)	1391 ± 501	
median (IQR) (ms)	1600 (751)	
NA, n (%)	69 (35)	

Abbreviations: *WHO* World Health Organization, *SD* standard deviation, *IQR* interquartile range, *PTBE* peritumoral brain edema, *EOR* extent of resection, *STR* subtotal resection, *GTR* gross total resection, *MRI* magnetic resonance imaging, *NA* not available

*Patient with multiple meningiomas with varying WHO grade

### Segmentation task

In the cross-validation dataset, the tumor segmentation model achieved a mean Dice coefficient of 0.87 ± 0.23, a mean Jaccard index of 0.82 ± 0.23, and a mean HD95 of 6.38 ± 20.66 mm. In the external validation dataset, the corresponding scores were 0.86 ± 0.17, 0.79 ± 0.18, and 6.20 ± 13.03 mm, respectively.

**Fig. 1:**
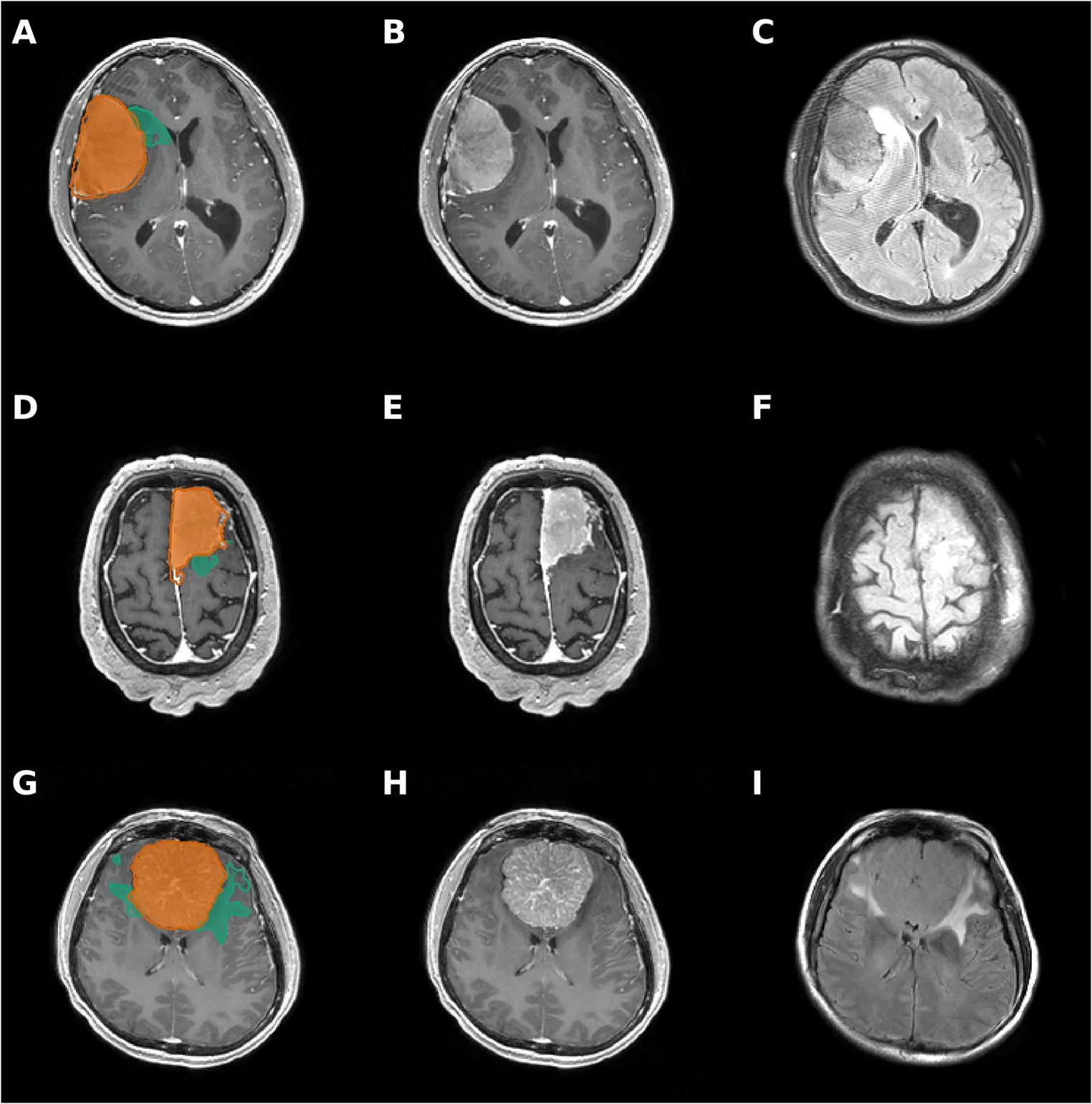
Representative examples of automated meningioma segmentation across three tumors on axial MRI. Each row corresponds to a different patient. From left to right, columns show: contrast-enhanced T1-weighted images with predicted segmentations of the tumor (orange) and peritumoral edema (green), the corresponding unannotated contrast-enhanced T1-weighted images, and FLAIR images highlighting the edema. The examples include three different cases (**A**–**C**), (**D**–**F**), and (**G**–**I**)

**Fig. 2:**
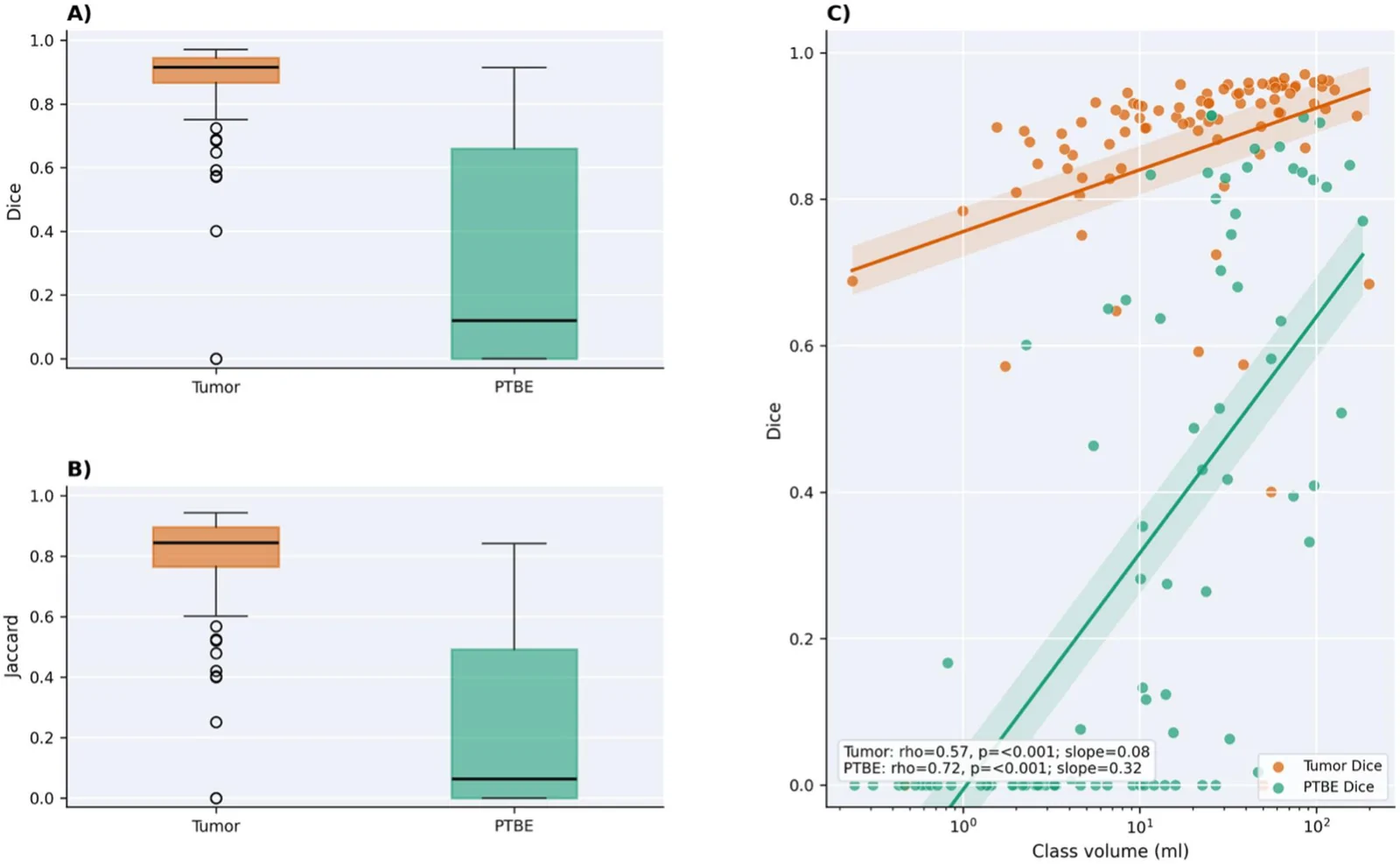
Metric performance on the external dataset. **A**: Boxplots of Dice scores of tumor and PTBE on the entire external dataset. **B**: Boxplots of Jaccard scores of tumor and PTBE on the entire external dataset. **C**: Scatterplot of Dice score and label volume on a logarithmic scale. Further, linear regression analysis with 95% confidence band with Spearman’s rho and *p*-values is displayed. Abbreviations: PTBE: peritumoral brain edema

For peritumoral brain edema segmentation, the model achieved a mean Dice coefficient of 0.63 ± 0.38, a mean Jaccard index of 0.55 ± 0.35, and a mean HD95 of 12.81 ± 34.90 mm in the cross-validation dataset. In the external validation dataset, the corresponding scores were 0.31 ± 0.35, 0.25 ± 0.30, and 22.30 ± 19.90 mm, respectively. Examples of the model-generated segmentations are shown in Fig. 1. Detailed model performance information is provided in Table 2. Figure 2 shows the distribution of performance on the external dataset and regression analysis of Dice score and label volume. Supplementary Figs. 1–4 illustrate further cross-validation performance and subgroup analysis.

**Table 2:** Performance of the nn-UNet during internal cross-validation on Zurich dataset and external validation on the TCIA dataset

	Cross-validation			External validation		
	Dice	Jaccard	HD95	Dice	Jaccard	HD95
Preoperative						
Tumor						
Mean ± SD	0.87 ± 0.23	0.82 ± 0.23	6.38 ± 20.66	0.86 ± 0.17	0.79 ± 0.18	6.20 ± 13.03
Median (IQR)	0.94 (0.05)	0.89 (0.09)	1.0 (1.0)	0.92 (0.08)	0.84 (0.13)	2.24 (2.71)
PTBE						
Mean ± SD	0.63 ± 0.38	0.55 ± 0.35	12.81 ± 34.90	0.31 ± 0.35	0.25 ± 0.30	22.30 ± 19.90
Median (IQR)	0.84 (0.70)	0.72 (0.72)	2.24 (4.20)	0.12 (0.66)	0.06 (0.49)	19.18 (22.18)

Abbreviations: *HD95* 95th percentile Hausdorff distance, *PTBE* peritumoral brain edema, *SD* standard deviation, *IQR* interquartile range

## Discussion

Meningiomas are the most common primary intracranial tumors worldwide [33, 34]. Due to their mostly slow growth, longitudinal imaging and accurate tumor volume assessment are critical for clinical monitoring [5, 35]. Currently, tumor size is commonly estimated through manual linear measurements or semi-automated volumetric tools, both of which are time-consuming and prone to variability [11, 12]. Furthermore, most existing segmentation models do not include peritumoral edema segmentation [24]. To address these limitations, we developed a fully automated, multi-channel deep learning model based on a 3D nnU-Net architecture for the segmentation and volumetric analysis of preoperative meningiomas and their associated peritumoral brain edema. The tumor model performed strongly on both the cross-validation and external validation sets, while the brain edema showed more variable performance, consistent with the greater complexity of the task.

While many meningioma segmentation models have demonstrated promising performance, several are limited by a lack of external validation and generalizability across datasets [22, 23]. A recent meta-analysis by Wang et al. pooled results from nine CNN-based studies involving 4,828 patients and reported a mean Dice of 0.89 (95% CI: 0.87–0.90). This is consistent with our external validation result of 0.86 ± 0.17. The analysis also found that models trained on multiple MRI sequences outperformed those using a single modality.

Among the nine studies included in the meta-analysis, Kang et al. implemented a 2D nnU-Net and achieved Dice scores of 0.922 and 0.893 for internal and external validation, respectively [36]. Their model was trained on a large cohort of 659 patients and employed extensive postprocessing, which may explain the strong performance despite its 2D design [36]. However, their reliance on T1CE images alone may limit clinical applicability due to variability in contrast enhancement patterns [36]. In contrast, our model incorporated both T1CE and FLAIR MRI sequences, allowing for more robust tumor characterization and enabling simultaneous assessment of contrast-enhancing tumor and peritumoral edema, both of which are relevant for clinical decision-making.

Although several additional meningioma models have been published since the development of our model, most have focused primarily on contrast-enhancing tumor segmentation and have not incorporated FLAIR-based peritumoral edema segmentation or edema segmentation more broadly [24]. Consequently, the simultaneous automated segmentation of both tumor and peritumoral edema remains an important novelty and clinically relevant strength of our approach. This is particularly relevant because peritumoral brain edema is often associated with greater symptom burden and an increased risk of postoperative complications in patients with intracranial meningiomas [37]. Peritumoral edema can also complicate surgical management and affect the safety of stereotactic radiosurgery [38]. In clinical practice, automated edema segmentation could support treatment selection and follow-up evaluation, particularly in radiosurgical workflows [39, 40]. While PTBE segmentation performance was lower in the external dataset overall, with a mean Dice score of 0.31 ± 0.35, performance varied by tumor location. In particular, PTBE segmentation was more accurate for convexity and parafalcine/parasagittal meningiomas, as shown in Supplementary Fig. 2. Furthermore, Supplementary Fig. 1C demonstrates that Dice scores increased with edema volume, indicating that larger edemas were segmented more accurately on average than smaller edemas. Overall, these findings indicate that automated PTBE segmentation performs best in selected anatomical locations and in patients with larger edema volumes, whereas smaller or anatomically more complex edemas may require closer manual review and correction.

Beyond structural MRI, molecular imaging and theranostic strategies are increasingly being explored in meningioma management [8, 9]. Somatostatin receptor-targeted PET/CT, most commonly using radiolabeled somatostatin analogs may provide complementary information to MRI [10]. In patients with sufficient receptor expression, PRRT represents an emerging targeted treatment option for recurrent or treatment-refractory meningiomas [10]. AI-driven segmentation could support these workflows by improving lesion delineation, treatment planning, and response assessment [41]. In the future, such approaches may be combined with radiomics, molecular imaging biomarkers, and emerging targeted or nanomedicine-based strategies to further personalize meningioma treatment.

From a practical implementation perspective, our model can be used through the publicly available graphical user interface (GUI) mentioned (Supplementary Fig. 5). Ideally, such a model would be integrated directly into the institutional imaging software to enable automated segmentation and volumetric measurements in the background. This would easily allow the surgeon or radiologist to review the segmentations, correct them if necessary, and incorporate the information into the radiology report or operative plan. Importantly, several factors may need to be considered for clinical implementation, including inference time, generalizability across heterogeneous imaging protocols, and the amount of manual correction required before the output can be used clinically [42]. Regarding inference time, it is reasonable to assume that hospitals will increasingly have access to local GPUs or secure APIs connected to high-performance GPUs, thereby enabling rapid inference in clinical workflows.

A model such as ours may be particularly useful in longitudinal follow-up of meningiomas, as reproducible tumor and edema measurements are crucial for detecting growth and assessing treatment response [5]. However, in its current form, the model was trained on images from single time points, and it remains unclear how reliably the model performs across longitudinal time series or detects minimal changes in tumor or edema volume. Future versions of the model should therefore be evaluated specifically for longitudinal follow-up to ensure reliable and standardized assessment over time.

Although newer segmentation architectures, including vision transformers (ViT) and diffusion-based models, have shown promise [43–48], the value they provide in relation to workflow feasibility must be considered. Increasing model complexity may improve segmentation performance but can also increase computational demand and latency, thereby hindering clinical deployment. In contrast, nnU-Net offers a pragmatic balance between performance and implementation feasibility, as it provides strong segmentation accuracy without extensive manual tuning and has been widely validated across medical imaging tasks [15–20]. For clinical deployment, this balance is likely more relevant than the marginal performance gains from more complex architectures.

## Limitations

Several important limitations of this study should be acknowledged. First, the manual annotations used as ground truth in both datasets are inherently subjective. In addition, formal interobserver agreement was not assessed, which further limits the reliability of the annotations. Second, the relatively modest sample size may have limited the model’s overall performance and generalizability. Third, although the model showed promising performance, we did not formally evaluate inference time, or the amount of manual correction required after automated segmentation. These factors are highly relevant for clinical translation and should be assessed in reader studies. Finally, as with all deep learning approaches intended for clinical use, several additional steps are necessary before successful clinical translation. These include model refinement, further external validation, prospective validation, reader studies, and integration into existing clinical workflows and information systems. Future studies should evaluate not only segmentation accuracy but also clinical usability and the potential impact of automated segmentation on treatment planning and follow-up decisions.

## Conclusion

We developed and validated a multichannel 3D nnU-Net model for the automated segmentation of meningiomas and peritumoral edema. The model showed strong segmentation performance for tumor and modest performance for edema segmentation, respectively. Future work should focus on broader validation, with a potential focus on the performance of the model across different tumor localizations, as well as prospective evaluation to support clinical adoption.

## Supplementary Information

Below is the link to the electronic supplementary material.

Supplementary Material 1

## Data Availability

The data in support of our findings can be obtained upon reasonable request from the corresponding author.

## Declarations

### Generative AI and AI-assisted technologies in the writing process

During the preparation of this work the authors used ChatGPT-4o (Open AI, San Francisco, CA) to receive suggestions for improving sentence structure and word choice. After using this tool, the authors reviewed and edited the content as needed and take full responsibility for the content of the published article.
