# Supplementary material for: Automated deep learning–based segmentation and volumetric analysis of meningiomas
Source: Neuroradiology. 2026 Aug 20;68(9):2917–26. doi: 10.1007/s00234-026-04152-z (PMC13680523; doi:10.1007/s00234-026-04152-z)
Supplement: Supplementary file 1 [file 234_2026_4152_MOESM1_ESM.docx]

***Supplementary Material***

**Supplementary Figure 1**. Cross-validation performance across the five folds on the 20 validation cases. Cross-validation performance of each of the five folds on the 20 validation cases.

A: Boxplots of Dice scores of tumor and PTBE across the five folds.

B: Boxplots of Jaccard scores of tumor and PTBE across the five folds.

C: Scatterplot of Dice score and label volume for tumor and PTBE segmentations.

***Abbreviations:*** *PTBE: peritumoral brain edema*

**Supplementary Figure 2**. External validation performance stratified by subgroups.

A: Boxplots of Dice score for tumor and PTBE stratified by histological WHO grading.

B: Boxplots of Dice score for tumor and PTBE stratified by tumor location.

***Abbreviations:*** *PTBE: peritumoral brain edema*

**Supplementary Figure 3**. External validation performance stratified by subgroups.

A: Boxplots of 95^th^ percentile Hausdorff Distance for tumor and PTBE stratified by histological WHO grading.

B: Boxplots of 95^th^ percentile Hausdorff Distance for tumor and PTBE stratified by tumor location.

***Abbreviations:*** *PTBE: peritumoral brain edema; HDD95: 95^th^ percentile Hausdorff Distance*

**Supplementary Figure 4**. External validation performance stratified by subgroups.

A: Boxplots of Jaccard score for tumor and PTBE stratified by histological WHO grading.

B: Boxplots of Jaccard score for tumor and PTBE stratified by tumor location.

***Abbreviations:*** *PTBE: peritumoral brain edema;*

**Supplementary Figure 5**. Example of a case displayed in the graphical user interface (GUI). The GUI is available at: <https://github.com/MICN-Lab/Meningioma_Segmentation/>.
